# Individual, community and health systems factors influencing time to notification of tuberculosis: situating software and hardware bottlenecks in local health systems

**DOI:** 10.1186/s12913-024-11697-3

**Published:** 2024-10-16

**Authors:** Sandra Beauty Chilala, Adam Silumbwe, Joseph Mumba Zulu, Moses Tetui, Maio Bulawayo, Mwimba Chewe, Peter Hangoma

**Affiliations:** 1 Department of Health Policy and Management, School of Public Health, University of Zambia, Lusaka, Zambia; 2 Department of Epidemiology and Global Health, Umeå University, 901 87 Umeå, Sweden; 3 Chr. Michelson Institute (CMI), P.O. Box 6033, Bergen, N-5892 Norway; 4 Bergen Centre for Ethics and Priority Setting in Health (BCEPS), University of Bergen, P.O. Box 7804, Bergen, N-5020 Norway; 5 School of Public Health Sciences, University of Waterloo, Waterloo, ON Canada; 6 School of Pharmacy, University of Waterloo, Kitchener, ON Canada

**Keywords:** Community, Detection, Factors, Hardware, Health systems, Software, Tuberculosis, Zambia

## Abstract

**Background:**

Despite several global interventions, tuberculosis (TB) remains a leading cause of death affecting millions of people globally. Many TB patients either have no access to quality care or go undetected by national health systems. Several multilevel factors account for under-detection of persons with TB. This study sought to explore patient-related software, community and health systems software and hardware factors influencing time to notification of TB in Lusaka District, Zambia.

**Methods:**

This was an exploratory qualitative case study that adopted a software and hardware lens of conceptualizing health systems. Data were collected from across three sites – urban and peri-urban areas: Chongwe, Kafue, and Lusaka – within Lusaka Province, Zambia. Sixteen key informants - TB corner nurses, community TB treatment supporters, and TB program managers - were interviewed. Six focus groups were held with TB patients. Data were analyzed using thematic analysis.

**Results:**

The study identified factors influencing timely TB notification, categorized into software and hardware elements.

Patient-related software elements, including TB knowledge and awareness, and health-seeking behavior, are crucial for prompt notification among TB patients.

In the community health system, software elements like social stigma and undesirable community attitudes towards contact tracing, and hardware elements such as unbalanced schedules, excessive workload and limited capacity of community TB treatment supporters contribute to delayed TB notification.

In the formal health system, software elements like negative attitudes of health providers towards TB patients and demotivation of TB staff, and hardware elements such as high diagnostics and transportation costs, outdated diagnostics in primary care facilities, and slow referral mechanisms, can also delay TB notification.

**Conclusion:**

Delays in time to TB notification are influenced by a combination of software (attitudinal and behavioral) and hardware (resource-related) elements across TB patients, community health systems, community TB treatment supporters, health providers, and TB staff. Addressing these factors, particularly social stigma, negative attitudes, and resource constraints, is crucial to improving timely TB detection and treatment.

**Supplementary Information:**

The online version contains supplementary material available at 10.1186/s12913-024-11697-3.

## Background

Tuberculosis (TB) remains a leading cause of death worldwide, claiming 1.6 million lives in 2021 [1]. The COVID-19 pandemic has exacerbated the TB crisis, undoing years of progress in prevention and treatment efforts [2]. Notably, 2020 saw a significant surge in TB-related deaths, the first increase in over a decade [3]. Furthermore, the number of newly diagnosed and reported persons with TB plummeted from 7.1 million in 2019 to 5.8 million in 2020, falling significantly short of the estimated 10 million people who contracted TB in 2020 [1].

The World Health Organization’s (WHO) End TB Strategy aims to eradicate TB by 2030, with a core focus on systematic screening and treatment for individuals at risk [4]. Achieving this goal relies on enhancing TB diagnosis and treatment for those seeking healthcare with symptoms (passive case finding) and proactively screening and caring for high-risk populations (active case finding) [5]. However, according to the WHO, a significant gap persists, with approximately 4.1 million people with TB remaining undiagnosed and unreported to national TB programs in 2020 [4].

Delays in proactive TB detection can be attributed to various health system factors, including the unavailability of modern diagnostic tools, human resource shortages, and inadequate policy reforms [6, 7]. Furthermore, individual and community health system factors - encompassing local networks, relationships, and processes that promote and support health - also contribute to these delays [8]. For instance, distrust in the health system, stigma, discrimination, and self-medication are common practices at the individual and community levels that often lead to delayed TB care-seeking [9–11]. These delays not only hinder timely diagnosis and treatment but also facilitate transmission within healthcare facilities, households, and communities. Moreover, delayed diagnosis exacerbates the disease, leading to additional complications and higher mortality rates [10].

In Zambia, TB continues to pose a substantial challenge to the health system, mirroring the situation in many other low- and middle-income countries [1]. In 2017, over 62,000 individuals contracted TB, making it one of the most heavily burdened nations worldwide [12]. With a global ranking of 13th in TB prevalence, Zambia faces a substantial burden, exacerbated by a high HIV co-infection rate of over 70% among TB patients [3]. Further, the 2013–2014 TB national survey revealed a twofold higher prevalence in urban compared to rural areas, disproportionately affecting the lowest wealth quintiles, particularly those in peri-urban areas [13]. However, only half of these (36,010) were officially reported, leaving the remaining half undiagnosed and unreported [12].

Encouragingly, TB notifications increased by 25% from 40,726 in 2020 to 50,825 in 2021 [14]. Zambia has also made progress in reducing TB mortality rates, from 228 per 100,000 populations in 2000 to 86 per 100,000 in 2020 [15]. Moreover, after peaking in 2019 and 2020, TB deaths declined in 2021 to their lowest level in over a decade [16].

Despite significant progress in reducing the TB burden, the disease continues to pose a major challenge to local health systems. Zambia has implemented various prevention, screening, and treatment initiatives to combat the TB pandemic [17]. However, the effective implementation of these programs has been hindered by several factors within local health systems, leading to suboptimal health outcomes [18]. Notably, the high percentage of unreported persons with TB and low treatment success rates have seen minimal improvement over the past decade [19]. Furthermore, weaknesses in diagnosing and treating people with suspected TB in some health facilities, coupled with fragile supply chains and inadequate infrastructure, have contributed to the spread of TB in clinical settings and communal environments [12, 20].

Understanding how health systems, community and individual levels factors influence delays in TB detection is critical, as early diagnosis and prompt treatment initiation are essential for effective TB programs [21]. In settings like Zambia, where most TB transmission occurs within households and communities [22], community health systems play a vital role in leveraging resources to generate demand for TB interventions [23]. The success of community-based TB interventions relies on both formal and community health systems factors, as highlighted in a growing body of literature [24, 25]. Specifically, community health workers within community health systems have been shown to enhance proactive TB detection and treatment success [26, 27]. Moreover, investing in TB diagnostic equipment has contributed to improved treatment success in many countries [28].

Therefore, this study sought to explore patient-related software, community and health systems software and hardware factors influencing time to notification of TB at health facility level in Lusaka District, Zambia.

### Hardware and software conceptualization of health systems

**Fig. 1:**
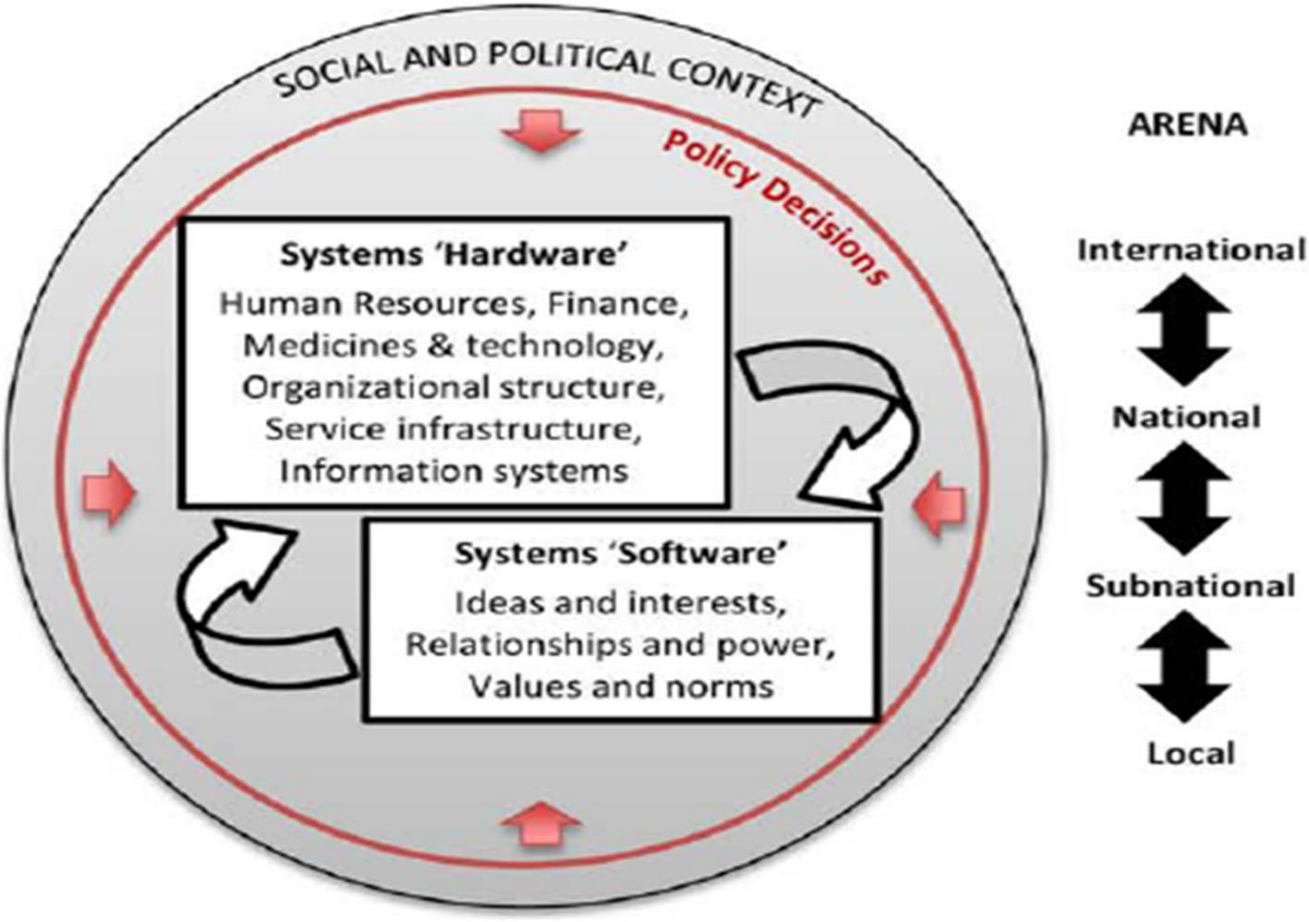
Health system conceptualization framework [30]

To understand the community and health system factors contributing to delays in TB detection, this study conceptualizes the health system in functional terms, comprising essential components like financial resources, medical products, information systems, human resources, service delivery, and governance [29]. Additionally, the study explores the “software” aspects, including ideas, interests, behaviors, attitudes, values, norms, and power dynamics that influence relationships and actions among system actors. At the community level, the health system is viewed as a network of local actors, relationships, and processes that produce, advocate for, and support health, while interacting with established health structures. This framework recognizes that health systems are socially and politically constructed, shaped by cultural factors and governance levels (community, health facility, and beyond) that impact the TB care cascade [30]. By adopting this framework, this study was able to comprehensively explore both the tangible and intangible factors affecting time to TB notification as well as assess the performance of local health systems with regards to TB services [30] (Fig. 1).

## Methods

### Study design

We conducted a qualitative case study to explore specific factors influencing time to TB notification in Lusaka, Zambia [31]. This in-depth case examination focused on urban and peri-urban health facilities and their surrounding communities, applying a community and health systems lens. We followed the COREQ guidelines in reporting the study findings (Supplementary file).

### Study setting

Zambia has a population of approximately 19 million, with a youthful demographic, as nearly half of the population is under the age of 15 [32]. The country faces significant socio-economic challenges, with over 50% of the population living below the poverty line, particularly in rural areas. Life expectancy is around 64 years, impacted by high rates of communicable diseases, malnutrition, and limited access to healthcare [33]. While HIV/AIDS remains a major health concern, Zambia grapples with a growing burden of non-communicable diseases. We conducted this study in six facilities in Lusaka, Zambia’s capital, which had the country’s second-highest TB prevalence (932/100,000) in 2013 [Ministry of Health report]. We selected three town areas with high TB diagnoses and diverse urban/peri-urban populations: Kafue Town (2 clinics), Chongwe Town (2 clinics), and Lusaka Town (2 hospitals).

### Study population, selection and recruitment of participants

The study population included health service providers (TB treatment supporters, community workers, program managers) and TB patients (≥ 18 years) from selected facilities and their surrounding areas.

We used purposive sampling to select participants, targeting healthcare providers and treatment supporters working in TB corners and outpatient departments [34]. Facility managers directed us to potential participants, including TB staff and confirmed TB patients identified through registers.

### Data collection

We collected data through 16 key informant interviews (KIIs) and 6 focus group discussions (FGDs) using guides developed from the literature review (Appendix 1 and 2). Although data saturation was reached after 14 KIIs and 5 FGDs, we conducted additional interviews to confirm findings and ensure credibility.

The principal researcher scheduled interviews with participants via phone calls, assisted by health facility and community leadership. All data were collected in English, audio recorded, transcribed verbatim, and supplemented with field notes. Two trained research assistants piloted the tools before data collection.

A total of 16 KIIs took place at the TB corners and lasted between 30 and 40 minutes. The distribution of the interviewees and their roles is indicated in Table 1.

**Table 1:** Key informant interviews

Category of participants	Role	Number interviewed
TB corner nurse	Supervise treatment supporters	6
TB treatment supporters	Screening and identifying people for TB care	6
TB program managers	Facility TB programing	4
Total number of KIIs		**16**

We conducted 6 FGDs, 2 in each of the 3 town areas, with 8 TB patients from each facility’s catchment population, totaling 48 participants (Table 2). To enrich perspectives, FGDs included both males and females, as delayed TB detection is not a sensitive topic [35]. The first author facilitated the discussions, assisted by trained research assistants who took notes. FGDs were conducted in the local language of Nyanja, audio-recorded, translated and transcribed verbatim.

**Table 2:** Focus group discussion

Category of Participants	Number FGDs	Age Range	Sex	Area Type	Number of Participants
Chongwe town	2	> 18	Male& Female	Peri-urban	16
Kafue town	2	> 18	Male& Female	Urban	16
Lusaka main	2	> 18	Male& Female	Urban	16
Total number of	**6**				**48**

### Data analysis

We employed thematic analysis to identify patterns and relationships in the qualitative data, exploring similarities and differences across subgroups, such as healthcare providers and patients [36].

Inductively, the process involved: familiarization: Reviewing field notes, observations, and coding transcripts into smaller units. Theme development: Collaborative discussions with the research team to merge and collapse codes into themes. Deductively, the inductively developed themes were applied to Fig. 1’s software and hardware elements of the health system leading to the final themes as indicated in Table 3.

**Table 3:** Thematic areas

Main themes	Sub-themes
▪ Patient related software	▪ TB Knowledge and awareness
	▪ Health seeking behavior
▪ Community health system software	▪ Social stigma against TB patients
	▪ Community attitudes towards contact tracing
▪ Community health system hardware	▪ Scheduling of TB treatment supporters ▪ Training of TB treatment supporters
▪ Formal health systems software	▪ Health provider attitudes ▪ Motivation of TB staff
▪ Formal health systems hardware	▪ Diagnostic and transportation costs
	▪ Diagnostics in primary care
	▪ Referral mechanisms from primary to secondary care

### Ethical consideration

This study received ethical approval from the University of Zambia Biomedical Research Ethics Committee (REF.No.314–2019) and Zambia National Health Research Authority. We obtained permissions from provincial and district health offices and ensured written informed consent from participants. To maintain privacy, we used participant identifiers instead of actual names and stored data securely on a password-protected computer. We minimized risks by conducting FGDs in open-air TB corners, avoiding transmission concerns.

## Results

In terms of patient-related software factors, the study identified TB knowledge and awareness, health-seeking behavior, while for community health system, software included social stigma and community attitudes towards contact tracing as factors influencing time to TB notification. Meanwhile, hardware elements within the community health system such as scheduling and training of TB treatment supporters also shaped time to TB notification.

With regards to the formal health system, health provider attitudes and motivation of TB staff were identified as software elements influencing time to TB notification. Additionally, hardware elements shaping time to TB notification in the formal health system comprised diagnostics and transportation costs, availability of diagnostics in primary care facilities, and referral mechanisms.

### Patient-related software elements influencing time to TB notification

#### TB knowledge and awareness

Most of the TB patients held varying perceptions about the disease. Some mentioned learning about it from community TB treatment supporters. Others were aware about TB being airborne and emphasized the importance of practicing cough etiquette to prevent transmission. One male participant expressed as follows:

“I learned about TB when the treatment supporter came to visit my neighbor who had not been feeling well for some time. She didn’t give much detail about TB but what I heard was that it is an airborne disease and so when coughing, a person coughing is supposed to hold their mouth.” P6, FGD2, TB patient.

On the other hand, some TB patients admitted to not fully comprehending what TB entailed. They believed it could be contracted from excessive smoking and alcohol consumption, as well as from working in dusty environments. A good number struggled to differentiate TB symptoms from those of other respiratory tract infections. This confusion often led to delays in seeking care at healthcare facilities.

“…at first, I thought it was a mere cough and it would go away. I had body malaise; I was also coughing, and I was failing to breathe properly. I was also feeling tired all the time. I did not really know what I was suffering from until one treatment supporter who talked to me about TB symptoms advised that I to go to the clinic.” P8, FGD2, TB patient. 

#### Health seeking behavior

Some TB patients tended to seek care at health facilities only after a prolonged period of self-medication within the community. They typically did so when they observed that their symptoms were worsening. Due to the non-specific nature of TB symptoms and a belief in the efficacy of herbal remedies, some patients mentioned consulting traditional and spiritual healers initially. It was only after these alternative avenues proved ineffective that they eventually sought care at a health facility.

“I started with traditional medicine. Some people were telling me that maybe I had sex with a woman who is sick and now that cough. That’s how I decided to seek help from those who do herbal medicine; they provided some to me and gave me to drink. The cough did not go away, that is how I decided to go and buy drugs, and those drugs did not work for me, so I decided to come to the clinic.” P6, FGD6. TB patient.

Most TB patients would cough for a period ranging from two weeks to three months before visiting the health facility. They explained that they often waited in the hope of improvement doubting whether it was TB at all. One of them, who had been experiencing symptoms for six months, expressed the following sentiment:

“I was just suspecting that it was TB as it is the one with such symptoms, so it was in the range of 3months. I used to cough continuously, no matter how much I took cough medicine, there was no improvement, then I thought normal cough is not supposed to be like this, that is when I decided to visit the clinic.” P1, FGD2, TB patient.

Furthermore, some TB patients tended to discontinue their treatment prematurely upon noticing an improvement in their health, ultimately resulting in a relapse of the disease. These individuals would often hesitate to return to health facilities out of fear of being reprimanded by the healthcare providers. One TB patient who had ceased treatment expressed the following:

“…I got tired of taking the drugs, I felt like six months was a long time for me to continue. So, when I felt that all the symptoms were gone after three months of treatment, I decided to go on a break. A few months later, I fell ill again, and I had to start the treatment from scratch…” P3, FGD4, TB patient.

### Community health systems software elements influencing time to TB notification

#### Social stigma towards TB patients

According to the TB patients, when family, friends, and neighbors are aware that someone had TB, they often showed support by encouraging diligent adherence to medication rather than treating them differently. However, some community members did exhibit a change in behavior towards TB patients upon learning of their condition. This fear of potential ridicule leads some patients to take their medication secretly. Additionally, many relatives and neighbors mistakenly assume that TB patients are also HIV positive. Some TB treatment supporters shared that there is indeed stigma within the community, as their clients fear the judgment of their neighbors upon learning about their TB diagnosis. This stigma results in avoiding seeking care and struggling with treatment compliance.

“When my neighbor learned that I had been diagnosed with TB, she stopped all her children from playing with my children at our home. She said that her children would contract the disease. I really felt bad such that I had to report her to the TB treatment supporter so that she could talk to her, when she did, at least she allowed her children to play from our yard.” P7, FGD3, TB patient.

#### Community attitudes towards TB contact tracing

Several treatment supporters disclosed that tracking people with suspected TB in a peri-urban community posed a challenge, as some patients were hesitant to reveal their TB status. Some people with suspected TB provided incorrect contact numbers and/or residential addresses to conceal their condition from others. Ultimately, this contributed to delays in TB detection. A treatment supporter, who encountered resistance during home visits, shared the following:

“…patients will not give correct addresses to their homes and so it becomes difficult to make follow-ups while some even give wrong phone numbers. They do come to the health facility, and we even diagnose them with TB but when they go back to the community, they don’t want to be visited at home for fear of people knowing that they are TB patients…” TTS, KII2, treatment supporter.

### Community health systems hardware influencing time to TB notification

#### Scheduling of CHW work

The TB treatment supporters who are usually community health workers indicated that they are supposed to work 20% at the health facility and 80% in the community. The treatment supporters have an important role to ensure successful treatment of people with confirmed or suspected TB. They conduct outreach, TB education, informal counselling, social support, and advocacy as well as link patients to needed TB care services. However, the TB treatment supporters revealed that they worked 80% at the facility and 20% in the community due to the shortage of manpower at the facilities, particularly, in the peri-urban areas, which negatively affected their contact tracing schedules. One of the treatment supporters had this to say.

“What I know is that we are supposed to work 80% in the field and 20% here at the clinic. But it is difficult because we only have one TB nurse running the TB corner and so sometimes there is no one to attend to clients as they come here when the nurse is out on workshops for example.” TTS, KII5, treatment supporter.

#### Training of TB treatment supporters

Furthermore, some TB treatment supporters conveyed that they had been trained on TB/HIV co-infection management by several organizations including the Ministry of Health. However, some healthcare providers emphasized the need to do refresher courses for TB treatment supporters as it has been long since they underwent any such training. They explained that the new treatment supporters had not yet been trained but were merely using their experience to deal with TB patients. Additionally, the health providers indicated that staff from departments such as the out-patient needed refresher training to identify people with suspected TB because some were being missed and treated as mere cough. One health provider had this to say:

“…In as much as we have been trained, it has been five years since we trained on any TB topic. If it’s for our new treatment supporters, they have not had any sort of training since they joined us. Other departments also need this training because we miss a lot of cases just there at OPD.” KII TCN6, TB corner nurse.

### Health systems software influencing time to TB notification

#### Health provider attitudes

Most of the TB patients reported that the majority of health workers showed positive attitude and behavior towards them during their interactions. On contrary, a few argued that it depends on whom you find at the facility and providers tend to have different attitudes. They revealed that some providers lacked the skill to communicate with them, and were easily angered and shouted at them. According to the TB patients, provider attitude, could either discourage or encourage individuals to visit or not the health facility. One of the TB patients had this to say:

“They just treated us well, if that is how all health service providers used to work people would not be suffering when seeking healthcare. Others within the facility shout at patients (using bad language) but these here just work well. On my part, I didn’t have any problem being saved by them. So, both those that come to visit us in the community and those that serve us here just work well.” P7, FGD1, TB patient.

While one of those that mentioned negative attitude said this:

“Some healthcare providers do not know how to talk to patients in a polite and respectable manner. They sometimes shout at clients who come to the facility for no reason. They think we shall contaminate them with the disease (TB). They don’t even want to draw close to us when talking to us and we must get drugs through that small window.” P5, FGD5, TB patient.

#### Motivation of TB treatment support staff

The TB treatment supporters working from peri-urban clinics of Lusaka indicated that in the past, they were paid a daily allowance by the facilities, but that this was no more. However, those who were working from Lusaka urban revealed that they were still receiving a monthly allowance from some projects. Initially, most treatment supporters were provided with cooler boxes, gumboots, and umbrellas when they started their work. Additionally, those working in peri-urban areas were given bicycles. Nevertheless, these incentives have dwindled with the conclusion of funding from certain projects. The irregular provision of incentives and varying levels of remuneration have led to demotivation resulting in some treatment supporters not showing up for work and struggling to maintain consistent TB notifications and follow-up of people with suspected TB. One treatment supporter shared their perspective:

“At first when we just started working as treatment supporters, we used to receive K40 as a daily allowance. We were also given things to use while in the field, things like umbrellas, gumboots, cooler boxes to carry samples and so on but it has been long, and these things have since worn out and now we have nothing to use. We used to have funders like CIDRZ and CDC who were answerable to but now those projects came to an end.” TTS, KII2 treatment supporter.

### Health systems hardware influencing time to TB notification

#### Diagnostic and transportation costs

All the TB patients stated that certain TB drugs were provided free of charge. However, they mentioned having to pay for TB diagnostic services such as X-rays and lab investigations. Unfortunately, these services were often challenging to access in public health facilities due to a persistent shortage of reagents. Additional expenses related to treatment, such as purchasing food to support the medication process, were reported to cause delays in seeking TB treatment. Patients preferred to wait until they had raised the required amount to cover these service costs before visiting health facilities. One participant expressed their frustration with this situation.

“We do not pay any money for the drugs but only when we must go to the lab or use the X-ray which we 50 here at Chawama clinic. But even paying for this is hard because we have no money. I was told to eat heavy meals before taking the drugs as they are strong but where will I get the food? I have no strength to work anymore, and my wife also must take care of me, and she is not working. Our children are too small for them to bring money at home, it has not been easy.” P4, FGD3, TB patient.

Some TB patients shared that they incurred substantial expenses while traveling long distances to reach the health facility. They either walked or used public transport, especially when seeking drugs or laboratory services that were unavailable at the nearest facility. Some mentioned that they were referred to distant health facilities, prompting the extended travel. Additionally, some stated that many TB patients were too weak to cover long distances on foot to collect their medication, leading to treatment default or visiting the facility when the disease was advanced.

“For me to come to Chongwe health facility, I must cover a long distance and at some point, I must cross a stream. In the farms where I come from, we have no health facility. Treatment supporters are also unable to reach our homes because of the distance. I only get to meet them here at the facility. If I don’t have transport money, I must walk the distance. When I was very sick, it was difficult for me to come here to get drugs because my knees were very weak.” P6, FGD4, TB Patient.

#### TB diagnostics in primary care

Most of the health service providers revealed that they only had sputum smear (microscopy), which is a cost-effective tool for diagnosing TB and monitoring of treatment progress. However, they revealed that this tool has many drawbacks as sputum culture takes long to diagnose the TB infection which affects initiation of appropriate therapy. Also, it cannot easily detect TB in HIV infected individuals. Out of the six facilities that were visited, only two had both microscopy and Gene Xpert. Some providers indicated that they sometimes misdiagnosed people with suspected TB because of the type of diagnostics they used.

“Microscopy is what we use at this center. We don’t have a Gene Xpert machine here. For Gene Xpert, we send samples to Kafue Estate Clinic at times. Maybe some cases might be missed using microscopy because of the type of machinery used. If it is extrapulmonary TB, it can’t be detected using microscopy. We just refer such cases to Kafue District Hospital so that the doctors can do a further investigation because it is not only extrapulmonary TB that we send to the hospital.” KII-TCN4, TB corner nurse.

#### Referral mechanisms from primary to secondary care

Some healthcare providers explained that they refer patients to larger facilities when they are unable to make a TB diagnosis, either due to a lack of necessary tools or a shortage of specialized staff. This process can lead to delays in diagnosing TB, and it can also be financially burdensome for the patient, as they must restart the investigation process each time, they visit a new facility. In some instances, treatment supporters mentioned that they personally accompanied the patient to the referral hospital to ensure they are attended to. However, a few health providers mentioned that not all referred patients are followed up, especially if a patient decides not to go to the referred facility. One healthcare provider, who justified the necessity of referring TB patients due to their facility’s limitations, shared the following sentiment:

When we are unable to make a diagnosis on a patient, we refer them to a bigger facility. At this facility we do not have a doctor, so we must send them elsewhere, and sometimes the process takes long…” KII, TCN3, TB corner nurse.

Another healthcare provider narrated this:

“TB services refer patients to services providing HIV testing with or without subsequent HIV care. We have done this through modification of existing record-keeping systems and referral forms, and regular meetings of staff members to strengthen referral linkages. However, patients are lost in the process because of a lack of transportation. “KII-TCN 1, TB corner nurse.

## Discussion

This study explored the factors influencing the time to TB notification at health facilities in Lusaka District, Zambia, focusing on patient-related, community, and health systems aspects. The study found that patient-related software factors such as TB knowledge, awareness, and health-seeking behavior, impacted notification times. Within the community health system, social stigma and attitudes toward contact tracing were key software factors, while hardware elements like the scheduling and training of TB treatment supporters also played a role. In the formal health system, the attitudes and motivation of healthcare providers were important software issues, along with hardware factors such as diagnostics availability, transportation costs, and referral mechanisms.

### Patient-related software

Patient-related software factors, such as limited understanding of TB transmission, persisting beliefs associating TB with alcohol consumption and dusty environments, and stigma, impacted time to TB notification. Our research aligns with similar studies attributing TB transmission to factors such as genetics, alcohol consumption, and exposure to dusty environments [37, 38]. Participants in our study may have linked some of these activities to TB transmission due to the crowded nature of such environments, which often serve as fertile ground for transmission [39]. However, a noteworthy observation is that the participants did not seem to differentiate between mere exposure and actual TB transmission implying a limited understanding. This may possibly contribute to delays in seeking care, especially when individuals are uncertain about exposure to TB.

### Community health system software and hardware

Delays in time to notification among those with suspected TB often stem from the social stigma associated with the disease, particularly its link to HIV within communities. The fear of being judged, discriminated, or isolated from the community leads people with suspected TB to hesitate to seek medical attention [40]. This social stigma contributes to self-stigma, as individuals internalize negative societal attitudes, resulting in a feeling of shame [20, 41]. Further, the association of TB with HIV and AIDS exacerbates self-stigma, causing individuals who suspect TB to delay care by withholding critical information about their symptoms to avoid community scrutiny [9, 41, 42]. Additionally, this stigma-driven shame may explain why certain households obstruct health facility contact tracing efforts by providing incorrect contact details and addresses to the TB treatment supporters [43].

Unbalanced schedules and insufficient training for TB treatment supporters contribute to delays in time to notification of TB through several mechanisms as observed in other studies [44, 45]. Poor scheduling leads to missed follow-up appointments and targets, as well as delays in providing timely referrals for people with suspected TB [46]. This issue may arise from the conflicting workload demands of the TB treatment supporters’ responsibilities at both the facility and community levels. Additionally, inadequate training of TB treatment supporters may result in insufficient patient education on key aspects of TB transmission and symptoms, leading to the oversight of early TB signs and subsequent delays in TB notification [44].

### Formal health systems software and hardware

Negative attitudes among healthcare providers, such as irritability towards patients, remain a significant challenge affecting time to TB notification. Similar findings have been reported in other studies [9, 37]. A lack of sympathy from the providers often discourages individuals with suspected TB from openly discussing their symptoms and seeking medical care [47]. Additionally, these negative attitudes may also lead to low suspicion of TB, with providers dismissing symptoms and failing to recommend definitive diagnostic tests. Several factors contribute to these provider attitudes including overwhelming workloads that lead to frustration within the facilities [48].

Transportation and treatment costs also affect the time to TB notification. In many cases, people with suspected TB are responsible for covering the costs of certain treatments, including laboratory diagnostics, which often leads to delays in seeking care until they can gather the necessary funds [49]. Higher living costs in urban areas may further deter people with suspected TB from seeking timely care. Additionally, the availability of numerous public and private health facilities in urban/peri-urban settings can result in people with suspected TB visiting multiple facilities for diagnosis, incurring additional costs [50]. Navigating this complexity may lead to delays in time to TB notification because some facilities lack TB diagnostic capacity, and patients have to make several visits before being referred to other facilities [51].

Inadequate TB diagnostic capacity in public primary health facilities, especially when they rely heavily on sputum smear microscopy, contributes to delays in time to TB notification. Evidence shows that sputum examination and cultures lack the sensitivity to detect TB in its early stages, hindering timely diagnosis [37]. Additionally, the labor-intensive and time-consuming nature of these diagnostic methods further delays TB detection. In this study, only a few facilities utilized both sputum smear microscopy and GeneXpert technology. Although GeneXpert is more accurate for TB diagnosis, maintenance challenges tend to impair functionality, often leading to further delays in time to TB notification. The inadequate molecular TB diagnosis services may be partly attributed to lack of resources in a country like Zambia. A holistic health system approach is needed to develop molecular diagnostic capacity, encompassing human resource training, institutional capacity building, streamlined procurement and maintenance systems [52, 53].

### Strength and limitations

One of the main strengths of this study was the adoption of a systems lens, which provided valuable insights into how various software and hardware factors at patient, community and health systems levels influence time to TB notification. The iterative process of reading, revising, and discussing themes during data analysis and writing also increased the validity of our findings. Additionally, the diverse experience of the research team enriched the analysis and ensured accurate reporting of the results.

However, several limitations were identified. The lack of an estimate for the ‘delay in time to notification’ obscures the true extent of the issue. Data collection coincided with social unrest, marked by negative rumors about health research, leading to initial resistance from some participants. Coordinating the FGDs with TB-diagnosed individuals was particularly difficult, though participants eventually consented after extensive engagement. Additionally, the onset of the country’s first COVID-19 cases resulted in some key informant interviews being canceled.

## Conclusions

This study identified complex factors influencing time to TB notification in Lusaka District, Zambia. Patient-related software factors, such as limited TB knowledge and health-seeking behavior, and community software issues, like social stigma and negative attitudes towards contact tracing, contributed to delays. Hardware factors, including inadequate training and scheduling for TB treatment supporters and limited diagnostic capacity, also played a role. Within the formal health system, provider attitudes and motivation were identified as key software factors, while hardware elements like diagnostic and treatment costs, along with slow referral mechanisms, also contributed to delays in time to TB notification.

To address these challenges, tailored strategies are necessary, including training and awareness programs to tackle software issues, investment in diverse diagnostic technologies and training for healthcare staff, and streamlining healthcare services to reduce transportation and treatment cost barriers. By addressing these factors, Zambia can reduce delays in time to TB notification, improve health outcomes, and progress towards achieving the End TB Strategy goals.

## Supplementary Information

Supplementary Material 1

Supplementary Material 2

## Data Availability

The raw data analyzed in the current study are available from the corresponding author upon reasonable request.

## Abbreviations

TB: Tuberculosis
WHO: World Health organization
FGD: Focus group discussion
KII: Key informant interviews
