# Supplementary material for: Individual, community and health systems factors influencing time to notification of tuberculosis: situating software and hardware bottlenecks in local health systems
Source: BMC Health Serv Res. 2024 Oct 16;24:1241. doi: 10.1186/s12913-024-11697-3 (PMC11481775; doi:10.1186/s12913-024-11697-3)
Supplement: Supplementary file 2 — Supplementary Material 2 [file 12913_2024_11697_MOESM2_ESM.docx]

## **Appendix 1: Focus Group Discussion Guide**

Discussion ground rules

In this discussion, there is no right or wrong answer. We are all free to express our views. People are encouraged to speak through the facilitator. We can only talk after one person has finished talking so as not to interrupt them. No side discussions will be allowed. Whatever we discuss today will remain confidential and protected. You are not obliged to answer all the questions in the guide. You are free not to answer any question you may consider risky to your own goals.

[Turn on the recorders]

I am the facilitator………………. interviewing FGD………………...Date…………. Start

time……. End time…….

Knowledge and aware of TB disease and treatment

- - Describe what TB is in your own understanding.
  - How would someone acquire TB? What causes it?
  - How would you know that someone has TB? What are the symptoms?
  - Describe, any, of your experiences with TB? How does one get diagnosed?
  - What kind of treatment do patients with TB at the facility?

Health Seeking Behavior

- In your community, where would you go for help with regards to TB care and what would be your reasons?
- What other forms of health care for TB patients are available in this community?
- What treatment options do people in this community mostly use and why?
- How long does it take a TB patient to visit other treatment options before going to the health facility?

Attitude and Perceptions

- How do community members relate to TB patients?
- And one’s relatives?
- Did they encourage you or help you in any way to visit the health facility?
- Is TB diagnosis an embarrassment to ones’ family?
- Do friends and neighbors know that they have TB?
- How do they treat them after knowing?

Quality Of TB Care

- If you have had TB before or any of your household members, can you explain approximately how long it took to be diagnosed after first developing the symptoms?
- When you or they started visiting the health facility, how long did it take before you were diagnosed?
- How much were you or they charged for the TB service at the facility?
- How much time was spent at the facility before diagnosis?
- Where do people get the information on TB symptoms?
- How would you describe the attitude of health workers?
- Do health worker visit regard about TB matters?
- What are some of the reasons that made you or any member of household not want to visit the health facility even when you/they noticed the TB symptoms?

Accessibility of TB Care

- Why do you come to this facility and not another?
- How do you travel to the facility?
- How long does it take you to reach the facility?
- How much does it cost you to get to the health facility?
- In your opinion, what can be done to encourage people who may have TB in the community to report to the health facility for detection?
- What could be some other causes of delay in seeking care among TB patients in the community?

**Thank you for your time**

## **Appendix 2: In-Depth Interview Guide**

TB Corner Nurse

- Describe your experience with handling TB cases?
- Are these TB followed up in the community to see if patients are taking their medication?
- Have you at any point been trained on TB awareness?
- What kind of technology do you use for TB diagnosis in this facility?
- What are the treatment methods available in the facility?
- Are there any CHWs that work with you in this facility, if so, how often do they go out in the communities to sensitize families on TB?
- How are they given the information to go and give out to communities?
- Afterward, have you observed an increase in the number of TB suspecting patients coming to the facility for diagnosis?
- Generally, what are the challenges that you have in early detection in TB as a facility?

Community health workers

- What available TB treatments do you know of?
- How was your training on TB-related ailments?
- Are there are any other groups that you work with within the community to teach families on TB, if yes, how do you work together?
- What is your experience working in the communities?
- What is your experience working with those from the health facility?
- Who is responsible for you and who do you report to?
- What incentives are there working with communities and the health facility?
- What are the challenges you have faced working in the community?

Program Managers

- What are the deliberate TB programs available for communities?
- How have communities worked with your organization
- What problems have you come across of why TB patients fail to seek care from health service providers?
- Could you describe challenges you face working with TB infested communities?
- How have worked around these challenges?
- Have you at any given point in time trained any group to help disseminate the TB awareness messages?

**Thank you for your time.**
